# Therapeutic efficacy of intra-articular adrenomedullin injection in antigen-induced arthritis in rabbits

**DOI:** 10.1186/ar2550

**Published:** 2008-11-13

**Authors:** Toshiyuki Okura, Kousuke Marutsuka, Hiroaki Hamada, Tomohisa Sekimoto, Tsuyoshi Fukushima, Yujiro Asada, Kazuo Kitamura, Etsuo Chosa

**Affiliations:** 1 Division of Orthopedic Surgery, Department of Medicine of Sensory and Motor Organs, Faculty of Medicine, University of Miyazaki, 5200 Kihara, Kiyotake, Miyazaki 889-1692, Japan; 2 Section of Pathophysiology, Department of Pathology, Faculty of Medicine, University of Miyazaki, 5200 Kihara, Kiyotake, Miyazaki 889-1692, Japan; 3 Section of Oncopathology and Regenerative Biology, Department of Pathology, Faculty of Medicine, University of Miyazaki, 5200 Kihara, Kiyotake, Miyazaki 889-1692, Japan; 4 Division of Circulatory and Body Fluid Regulation, Department of Internal Medicine, Faculty of Medicine, University of Miyazaki, 5200 Kihara, Kiyotake, Miyazaki 889-1692, Japan

## Abstract

**Introduction:**

Adrenomedullin is a potent vasodilatory and hypotensive peptide as well as an endogenous immunomodulatory factor with predominantly anti-inflammatory effects. The purpose of the present study was to evaluate the therapeutic effects of adrenomedullin in rabbits with antigen-induced arthritis, an experimental model of rheumatoid arthritis.

**Methods:**

Following the induction of arthritis in both knee joints by ovalbumin injection into the joint spaces of pre-immunized rabbits, increasing daily doses of adrenomedullin were injected into the knee joint spaces or saline was injected into the contralateral knee joint spaces as the control. For time-course experiments, adrenomedullin and saline were injected into the knee joint spaces daily for 7 days and 20 days. The degree of joint swelling and the histological change in the knee joints injected with adrenomedullin were compared with the control knee joints. Histological evaluation of the infrapatellar fat pads and synovial tissue was performed. TNFα, IL-6, vascular endothelial growth factor and transforming growth factor-beta mRNA levels in the synovial tissue were measured using real-time quantitative PCR.

**Results:**

Daily injections of adrenomedullin into the knee joint spaces of rabbits with antigen-induced arthritis decreased joint swelling. Histological examination revealed that adrenomedullin reduced edematous changes and the infiltration of inflammatory cells in the synovial tissues. Analysis of mRNA levels showed that adrenomedullin significantly reduced TNFα mRNA expression by 21% to 49% in a dose-dependent manner, and dose-dependently increased IL-6 mRNA expression by 45% to 121%.

**Conclusions:**

These results suggest that daily injections of adrenomedullin into the knee joint spaces of rabbits with antigen-induced arthritis ameliorated the inflammatory response in arthritic joints. Adrenomedullin may thus be useful as a treatment for rheumatoid arthritis; however, the effect of adrenomedullin on IL-6 production in the synovial tissue may be an undesirable adverse effect in rheumatoid arthritis therapy.

## Introduction

Rheumatoid arthritis (RA) is a chronic and systemic inflammatory disorder affecting multiple joints. The causes of RA are not fully understood, and the treatment has not been completely established. The cytokine network, consisting of many inflammatory cytokines, mediates the chronic inflammatory process, including that in RA. The balance between proinflammatory cytokines and anti-inflammatory cytokines is important in determining the grade and extent of inflammation. Considerable progress has been reported in the use of biological agents that mediate the pathogenesis of RA, especially antibodies to TNFα and soluble TNFα receptors [1,2].

Adrenomedullin (AM) is a 52-amino-acid peptide, which was originally isolated from extracts of human pheochromocytoma using elevated platelet cAMP activity as an indicator [3]. Besides its potent vasodilatory and hypotensive effects, AM is also known to have other multiple regulatory functions. Several studies have suggested that AM acts as an endogenous immunomodulatory factor, with predominantly anti-inflammatory effects. It has been reported that AM reduces the secretion of TNFα from activated macrophages [4-6]. In addition, AM has been shown to ameliorate colitis in murine models [7,8]. Moreover, AM was reported to abrogate arthritis in a murine model via an inhibitory effect on the T helper type 1-driven autoimmune and inflammatory responses [9].

We and other investigators have reported that elevated AM levels are found in plasma, joint fluid, and the synovium in RA [10,11]. From the observations of the anti-inflammatory effects of AM, it is speculated that the body responds to an inflammatory condition and attempts to ameliorate arthritis by increasing the secretion of AM.

The aim of the present study was to investigate the therapeutic effects of AM in an animal model of RA *in vivo*. We used rabbits with antigen-induced arthritis (AIA), an experimental model of RA [12,13]. We showed that daily injections of AM into the knee joint spaces of rabbits with AIA decreased joint swelling. Histological examination revealed that AM reduced edematous changes and the infiltration of inflammatory cells in the synovial tissues. Analysis of mRNA levels in the synovial tissue demonstrated that AM significantly reduced the TNFα mRNA level, but increased the IL-6 mRNA level. These results suggest that, although AM ameliorated joint pathology in the rabbit AIA model, the effect of AM on IL-6 production might be an adverse effect in RA therapy.

## Materials and methods

### Animals

Female Japanese white rabbits (Kyudo Co., Ltd, Saga, Japan) weighing 3.1 to 3.5 kg were used in the study. The rabbits were housed in a temperature-controlled and humidity-controlled room and were maintained on standard pellet chow and tap water. All experiments were performed under the regulations of the Animal Research Committee of Miyazaki University.

### Induction of antigen-induced arthritis

The AIA rabbit model was developed as described by Consden and colleagues [13]. Briefly, rabbits were anesthetized by an intravenous injection of pentobarbital sodium and were immunized by 1.2 ml intradermal injections of 6 mg/ml ovalbumin (Sigma-Aldrich, St Louis, MO, USA) in saline emulsified with an equal volume of TiterMax Gold (TiterMax, Norcross, GA, USA). The rabbits were re-immunized in the same manner 30 days later. Seven days after the second immunization, the rabbits underwent skin testing following a 0.1 ml intradermal injection of a solution of 200 μg/ml ovalbumin in saline. Animals exhibiting a welt of 13 mm or greater after 24 hours were confirmed as 'immunized'. Twelve days after the second immunization, the 'immunized' rabbits were anesthetized and arthritis was induced by 0.5 ml bilateral knee intra-articular injections of a solution of 20 mg/ml ovalbumin in saline.

### Treatment protocol

Twenty-four hours after arthritis induction, the rabbits were anesthetized and different doses of AM (1 ng to 3 μg; Peptide Institute Inc., Osaka, Japan) dissolved in 0.3 ml saline were injected into the knee joint spaces or 0.3 ml saline was injected into the contralateral knee joint spaces as controls. For time-course experiments, AM and saline were injected into the knee joint spaces daily for 7 days and 20 days. The rabbits were sacrificed on day 8 (*n* = 5 in each group) and day 21 (*n* = 3 in each group).

### Measurement of adrenomedullin in plasma

To evaluate the effect of intra-articular injection of AM on the blood concentration, whole-blood samples (total 1 ml) were taken from a peripheral artery in the rabbit ear using a 22-gauge needle before and 15, 30, 60 and 120 minutes after intra-articular injection of 3 μg AM. Blood samples were transferred into tubes containing 1 mg/ml disodium ethylenediamine tetraacetic acid and 500 kallikrein inhibitory units/ml aprotinin, and were centrifuged for 15 minutes at 1670 *g*. The plasma was stored at -30°C until assayed. Plasma AM concentration was measured using an immunoenzymometric assay kit [14].

### Joint swelling

To evaluate the grade of arthritis/inflammation, joint swelling was assessed by measuring the maximum diameter of the swollen joint using calipers. The swelling was compared with that at the same level on the contralateral knee, treated with saline.

### Histological evaluation

For histological evaluation, rabbits were given an overdose of pentobarbital 8 days and 21 days after arthritis induction. The infrapatellar fat pads were harvested from dissected knees and were cut longitudinally, perpendicular to the patella ligament in the middle of the infrapatellar fat pad. The tissues were fixed in 10% buffered formaldehyde and embedded in paraffin wax, and sections 3 μm thick were obtained. The specimens were stained with H & E and Mallory–Azan stains. The area of the infrapatellar fat pad was measured using AxioVision software (release 4.3; ZEISS, Oberkochen, Germany). Inflammatory cells, including lymphocytes and plasma cells, were counted in the superficial and deep portions of the infrapatellar fat pads (three fields under ×200 magnification in each portion) in H & E-stained specimens. The inflammatory cell count was performed by two independent observers.

To measure the collagen volume, the images of sections with Mallory–Azan stain were projected onto a color imaging analysis system (Mac SCOPE version 2.3.2; Mitani, Fukui, Japan). In each section, 10 separate sites were analyzed at ×40 magnification. The collagen volume fraction was obtained by calculating the mean ratio of connective tissue to the total tissue area.

### Measurement of cytokine mRNA

Total RNA was extracted from the infrapatellar fat pad with TRIzol reagent (Invitrogen, Carlsbad, CA, USA) according to the manufacturer's protocol and was then reverse-transcribed into cDNA with the SuperScript First-Strand Synthesis System for RT-PCR kit (Invitrogen). To measure rabbit TNFα, IL-6, vascular endothelial growth factor (VEGF), transforming growth factor beta (TGFβ), and β-actin mRNA levels, we used the quantitative RT-PCR method of real-time quantitative PCR.

Table 1 presents the sequences of the primers for TNFα [GenBank:M12845], IL-6 [GenBank: AF169176], VEGF [GenBank:AY196796], TGFβ [GenBank:AB020217], and β-actin [GenBank:AF309819] [15]. PCR was performed in a LightCycler (Roche, Basel, Switzerland) using the SYBR Premix Ex Taq kit (Takara Bio, Shiga, Japan) according to the manufacturer's instructions. We obtained data from three independent experiments. The mRNA levels were compared after they had been normalized relative to those of β-actin.

**Table 1:** Primers for real-time PCR

Gene	GenBank accession number	Product (base pairs)	Oligonucleotide sequences (forward and reverse primers)
TNFα^a^	[GenBank:M12845]	252	AGCCCACGTAGTAGCAAACCC
			TTGATGGCAGAGAGGAGGTTGA
IL-6	[GenBank:AF169176]	93	CCGGCGGTGAATAATGAGAC
			CCTGAACTTGGCCTGAAGGTG
Vascular endothelial growth factor	[GenBank:AY196796]	91	AATGATGAAAGCCTGGAGTGTGTG
			CTATGTGCTGGCCCTGGTGA
Transforming growth factor beta	[GenBank:AB020217]	136	AAGGACCTGGGCTGGAAGTG
			CCGGGTTGTGCTGGTTGTA
β-Actin	[GenBank:AF309819]	183	CCATGTACGTGGCCATCCAG
			TCTTCATGAGGTAGTCGGTCAGGTC

^a^Primer source was Reno and colleagues [15].

### Measurement of TNFα and IL-6

Protein extracts were isolated by homogenization of infrapatellar fat pads (50 mg tissue/ml) in 50 mmol/l Tris–HCl, pH 7.4, with 0.5 mmol/l dithiothreitol, and 10 μl/ml protease inhibitor cocktail (Sigma-Aldrich). The samples were centrifuged at 30,000 × *g *for 20 minutes and stored at -30°C until assayed. TNFα and IL-6 levels in the protein extracts were measured using ELISA kits for human TNFα and IL-6 (R&D Systems, Minneapolis, MN, USA) according to Zagariya and colleagues [16]. The TNFα level in the protein extracts was also measured by SDS-PAGE and western blotting using Armenian hamster anti-mouse TNFα monoclonal antibody (Santa Cruz Biotechnology, Santa Cruz, CA, USA). We could not, however, obtain worthwhile data by these methods (data not shown). It was considered that these ELISA kits and the anti-mouse TNFα monoclonal antibody may not cross-react with rabbit IL-6 and TNFα, or that TNFα and IL-6 levels in the protein extracts were lower than the detection limits of these assays.

### Statistical analysis

In all experiments, we compared values for AM-treated knees with control knees from the same animal. All data are expressed as the mean ± standard error. The differences were analyzed using the Mann–Whitney U test. *P *< 0.05 was considered statistically significant.

## Results

### Adrenomedullin concentration in plasma

**Figure 1:**
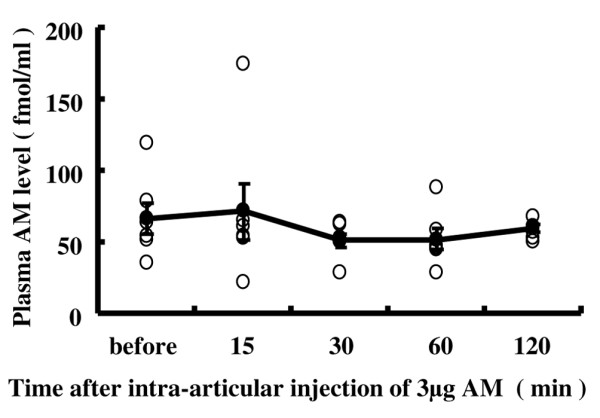
**Sequential concentrations of plasma adrenomedullin following intra-articular adrenomedullin injection in rabbits with antigen-induced arthritis**. Whole-blood samples (total 1 ml) were taken from a peripheral artery in the rabbit ear using a 22-gauge needle before and 15, 30, 60 and 120 minutes after intra-articular injection of 3 μg adrenomedullin (AM). The plasma AM concentration was measured using an immunoenzymometric assay kit (*n* = 6). White circles, plasma AM levels in rabbits; black circles, average plasma AM levels at each time point after intra-articular injection of 3 μg AM. Data expressed as the mean ± standard error of the mean.

We measured the plasma AM concentration before and 15, 30, 60 and 120 minutes after intra-articular injection of 3 μg AM (*n* = 6). No significant change, however, was observed in the plasma concentration of AM (Figure 1). The intra-articular injection of AM did not therefore increase the level of AM in plasma.

### Joint swelling

**Figure 2:**
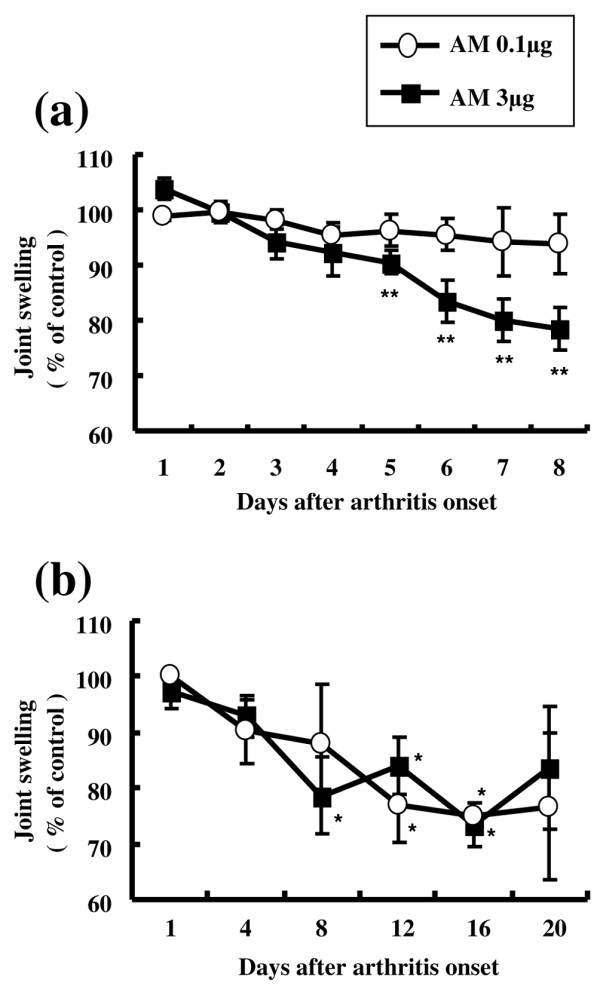
**Adrenomedullin reduced joint swelling in rabbits with antigen-induced arthritis**. Rabbits with antigen-induced arthritis (AIA) were treated with daily injections of adrenomedullin (AM) or saline (control) into the knee joint spaces beginning 24 hours after arthritis onset. Joint swelling was defined as the increase in knee diameter from normal and was compared with that at the same level on the contralateral knee, treated with saline. **(a) **Joint swelling progress in rabbits with AIA treated with daily intra-articular injections of AM or saline for 7 days (*n* = 5 in each group). Daily intra-articular injections of 3 μg AM significantly decreased joint swelling compared with contralateral knees after day 5. No significant decrease in joint swelling was observed in knees treated with <0.1 μg AM. **(b) **Joint swelling progress in rabbits with AIA treated with daily intra-articular injections of AM or saline for 20 days (*n* = 3 in each group). Daily intra-articular injections of 0.1 μg and 3 μg AM showed a tendency to reduce joint swelling throughout the experiment, and significantly decreased joint swelling on days 12 and 16 and on days 8, 12 and 16, respectively, compared with contralateral control knees. Daily intra-articular injections of 1 ng and 0.01 μg AM did not ameliorate joint swelling throughout the experiments (data not shown). Data expressed as the mean ± standard error of the mean. *P < 0.05 and **P < 0.01, compared with contralateral knees.

**Figure 3:**
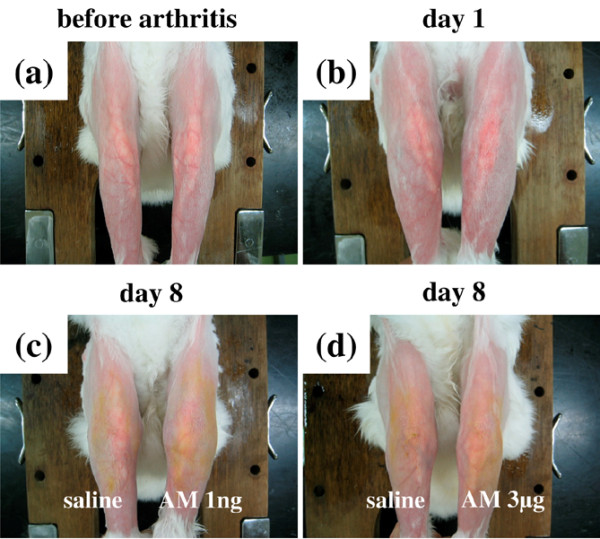
**Macroscopic pathology of joint swelling in rabbits with antigen-induced arthritis**. **(a) **Photograph taken before arthritis onset. **(b) **Photograph taken 24 hours after arthritis onset. **(c) **The left knee of the rabbit with antigen-induced arthritis (AIA) was treated with daily intra-articular injections of 1 ng adrenomedullin (AM) for 7 days and the right knee was treated with daily intra-articular injections of saline for 7 days. Photograph taken 8 days after arthritis onset. **(d) **The left knee of the rabbit with AIA was treated with daily intra-articular injections of 3 μg AM for 7 days and the right knee was treated with daily intra-articular injections of saline for 7 days. Photograph taken 8 days after arthritis onset.

To evaluate the anti-inflammatory effect of AM on arthritis, we used calipers to measure joint swelling in AM-treated knees and compared the swelling with that at the same level on the contralateral knees, treated with saline. In rabbits with AIA treated with daily injections of AM or saline into the knee joint spaces for 7 days, 3 μg AM significantly reduced joint swelling compared with contralateral knees after day 5. No significant decrease in joint swelling was observed, however, in knees treated with <0.1 μg AM (Figures 2a and 3). In rabbits with AIA treated for 20 days with daily injections of AM or saline into the knee joint spaces, 0.1 μg and 3 μg AM showed a tendency to reduce joint swelling throughout the experiment – and significantly decreased joint swelling on days 12 and 16 and on days 8, 12 and 16, respectively, compared with contralateral knees (Figure 2b). Daily intra-articular injections of 1 ng and 0.01 μg AM, however, did not ameliorate joint swelling (data not shown).

### Histological findings

**Figure 4:**
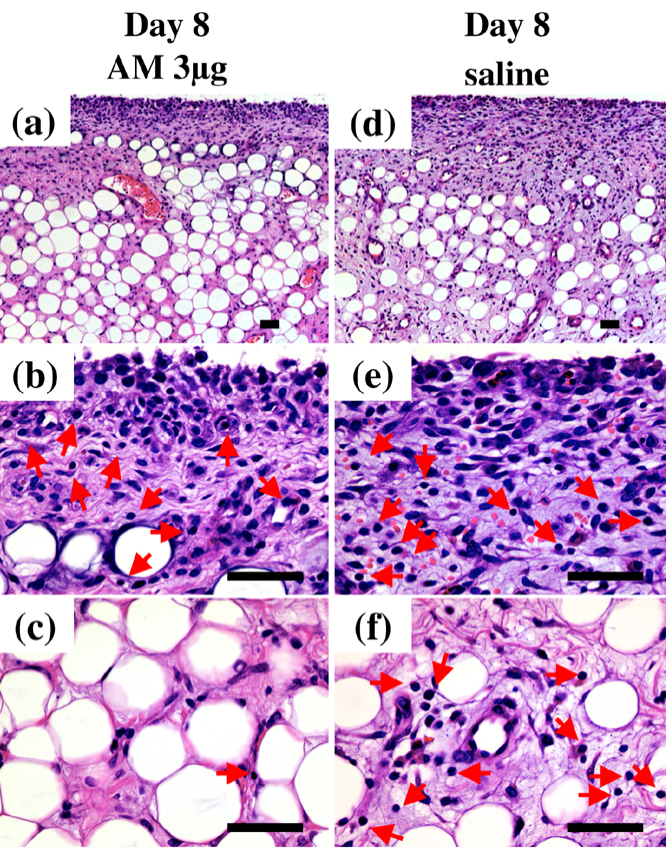
**Histological analysis of infrapatellar fat pad harvested from rabbit knees 8 days after arthritis onset**. Rabbits with antigen-induced arthritis (AIA) were treated with daily injections of adrenomedullin (AM) or saline (control) into the knee joint spaces for 7 days. The infrapatellar fat pads were harvested from rabbit knees 8 days after arthritis onset. Tissues were sectioned longitudinally perpendicular to the patella ligament in the middle of the tissue, and were stained with H & E. **(a)**, **(b)**, **(c) **AIA rabbit knee was treated with daily intra-articular injections of 3 μg AM for 7 days. (a) Low-magnification image (×100). (b), (c) High-magnification images (×400) of the superficial portion and the deep portion of (a), respectively. **(d)**, **(e)**, **(f) **The contralateral knee of (a), (b) and (c) was treated with daily intra-articular injections of saline for 7 days. (d) Low-magnification image (×100). (e), (f) High-magnification images (×400) of the superficial portion and the deep portion of (d), respectively. Arrows indicate inflammatory cells. Bar = 50 μm.

**Figure 5:**
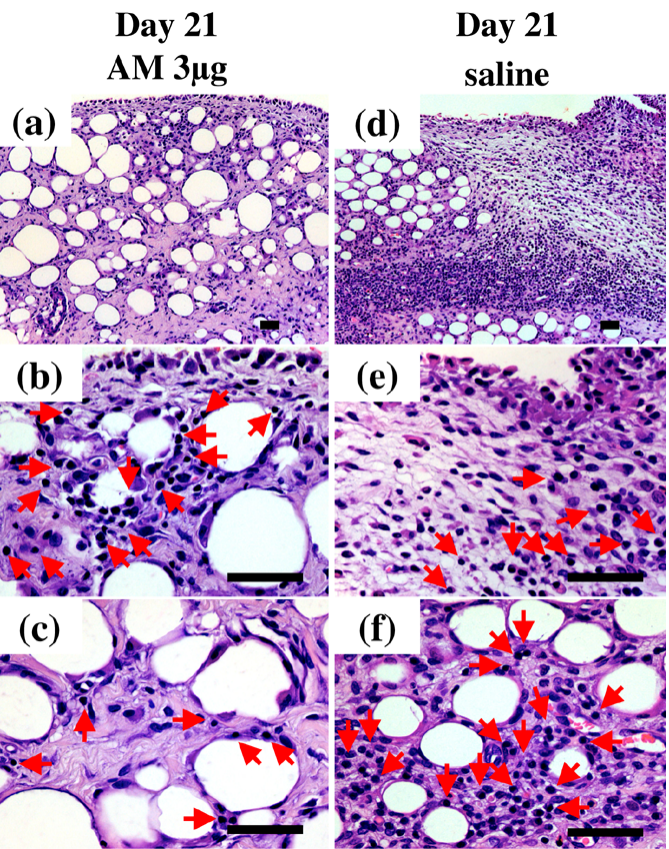
**Histological analysis of infrapatellar fat pad harvested from rabbit knees 21 days after arthritis onset**. Rabbits with antigen-induced arthritis (AIA) were treated with daily injections of adrenomedullin (AM) or saline (control) into the knee joint spaces for 20 days. The infrapatellar fat pads were harvested from rabbit knees 21 days after arthritis onset. The tissues were sectioned longitudinally perpendicular to the patella ligament in the middle of the tissue, and were stained with H & E. **(a)**, **(b)**, **(c) **AIA rabbit knee was treated with daily intra-articular injections of 3 μg AM for 20 days. (a) Low-magnification image (×100). (b), (c) High-magnification images (×400) of the superficial portion and the deep portion of (a), respectively. **(d)**, **(e)**, **(f) **The contralateral knee of (a), (b) and (c) was treated with daily intra-articular injections of saline for 20 days. (d) Low-magnification image (×100). (e), (f) High-magnification images (×400) of the superficial portion and the deep portion of (d), respectively. Arrows indicate inflammatory cells. Bar = 50 μm.

To evaluate the effect of AM on synovial tissue and intra-articular tissue in the inflamed joints, we examined the infrapatellar fat pads by histology. The infrapatellar fat pads harvested from control knees on day 8 showed a dense inflammatory reaction, including edematous changes in the synovial interstitium, intracellular edema in the infrapatellar fat pads, hyperplasia of synovial surface cells and widespread infiltration of inflammatory cells in the infrapatellar fat pads (Figure 4d,e,f). In contrast, these inflammatory reactions were suppressed in the knees treated with AM for 7 days. In particular, edematous changes in the synovial interstitium, intracellular edema in the infrapatellar fat pads and infiltration of inflammatory cells in the deep portion of the infrapatellar fat pads were significantly reduced (Figure 4a,b,c). The infrapatellar fat pads harvested from control knees on day 21 also showed a severe inflammatory reaction. Edematous changes in the synovial interstitium, hyperplasia of synovial surface cells and widespread infiltration of inflammatory cells throughout the infrapatellar fat pads were observed (Figure 5d,e,f). In the knees treated with AM for 20 days, these inflammatory reactions were ameliorated. AM treatment significantly suppressed infiltration of inflammatory cells in the deep portion of the infrapatellar fat pads (Figure 5a,b,c).

**Figure 6:**
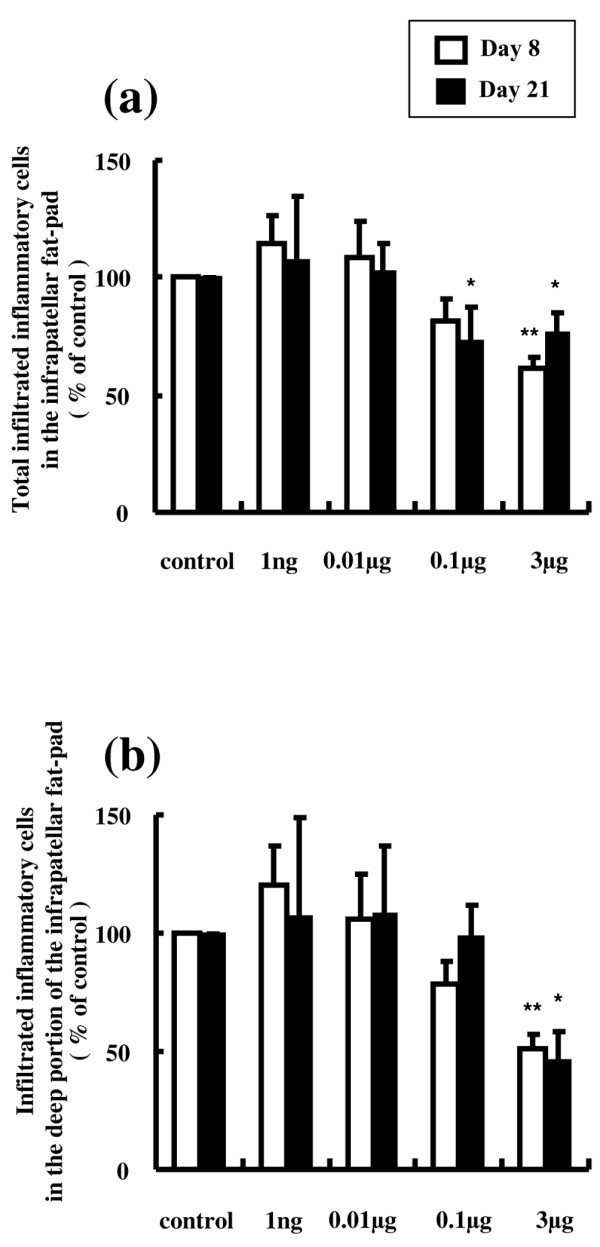
**Effect of adrenomedullin on the infiltration of inflammatory cells in the infrapatellar fat pad**. **(a) **Total number of inflammatory cells that infiltrated the infrapatellar fat pad (three sites in the superficial portion, three sites in the deep portion). The total number of inflammatory cells that infiltrated the infrapatellar fat pad was significantly reduced by 26% with daily intra-articular injections of 0.1 μg adrenomedullin (AM) on day 21. Daily intra-articular injections of 3 μg AM significantly suppressed the total number of inflammatory cells by 38% and 23% at day 8 and day 21, respectively. **(b) **Number of inflammatory cells that infiltrated the deep portion of the infrapatellar fat pad (three sites). Daily intra-articular injections of 3 μg AM significantly suppressed the infiltration of inflammatory cells in the deep portion of the infrapatellar fat pad by 49% and 54% at day 8 and day 21, respectively. Open and closed columns represent the data at day 8 (*n* = 5 in each group) and at day 21 (*n* = 3 in each group), respectively. Data expressed as the mean ± standard error of the mean. *P < 0.05 and **P < 0.01, compared with contralateral knees.

The total number of inflammatory cells that infiltrated the infrapatellar fat pad was significantly reduced by 26% at 0.1 μg AM on day 21 (Figure 6a). Daily intra-articular injections of 3 μg AM significantly suppressed the total number of inflammatory cells infiltrating the infrapatellar fat pad by 38% and 23% at day 8 and day 21, respectively, and suppressed the infiltration of inflammatory cells in the deep portion of the infrapatellar fat pad by 49% and 54% at day 8 and day 21, respectively, compared with the controls (Figure 6).

**Figure 7:**
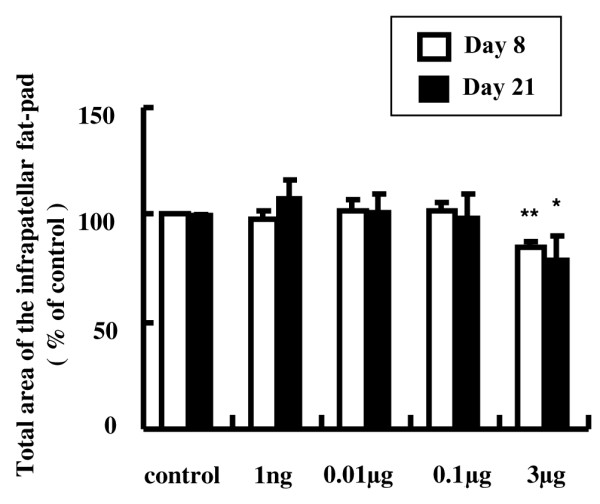
**Effect of adrenomedullin on the total area of the infrapatellar fat pad**. The infrapatellar fat pads were sectioned longitudinally perpendicular to the patella ligament in the middle of the tissue, and the total tissue area was determined using software. Daily intra-articular injections of 3 μg adrenomedullin (AM) significantly reduced the total tissue area by 15% and 20% at day 8 and day 21, respectively. Open and closed columns represent the data at day 8 (*n* = 5 in each group) and day 21 (*n* = 3 in each group), respectively. Data expressed as the mean ± standard error of the mean. *P < 0.05 and **P < 0.01, compared with contralateral knees.

To examine the effect of AM on tissue edema, we measured the total area of the infrapatellar fat pad using software. Daily intra-articular injections of 3 μg AM significantly decreased the total area of the infrapatellar fat pad by 15% and 20% at day 8 and day 21, respectively, compared with the controls (Figure 7).

**Figure 8:**
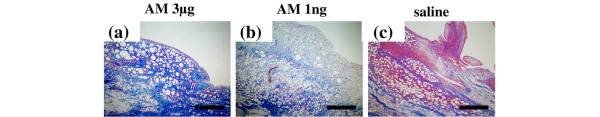
**Histological analysis of infrapatellar fat-pad sections stained with Mallory – Azan from rabbits with antigen-induced arthritis**. Rabbits with antigen-induced arthritis (AIA) were treated with daily injections of adrenomedullin (AM) or saline (control) into the knee joint spaces for 20 days. The infrapatellar fat pads were harvested from rabbit knees 21 days after arthritis induction. The tissues were sectioned longitudinally perpendicular to the patella ligament in the middle of the tissue, and were stained with Mallory – Azan. **(a) **AIA rabbit knee was treated with daily intra-articular injections of 3 μg AM for 20 days. **(b) **AIA rabbit knee was treated with daily intra-articular injections of 1 ng AM for 20 days. **(c) **The contralateral knee of (a) was treated with daily intra-articular injections of saline for 20 days. Photographs taken at ×40 magnification. Bar = 500 μm.

**Figure 9:**
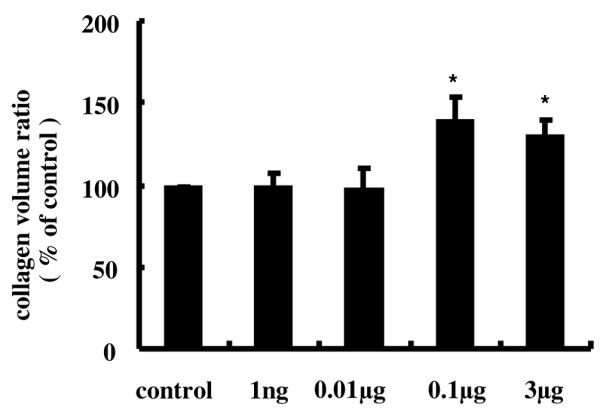
**Quantitative evaluation of collagen volume in the infrapatellar fat pad**. To measure the collagen volume, the sections with Mallory – Azan stain were projected onto a color imaging analysis system. In each section, 10 separate sites were analyzed and the collagen volume fraction was obtained by calculating the mean ratio of connective tissue to the total tissue area. The collagen volume ratio was increased in adrenomedullin (AM)-treated knees by 39% and 31% at 0.1 μg and 3 μg AM, respectively. Data expressed as the mean ± standard error of the mean. *P < 0.05, compared with contralateral knees.

To observe the effect of AM on fibrosis of the infrapatellar fat pads harvested on day 21, we examined the collagen volume ratio of the infrapatellar fat pad histologically using Mallory–Azan staining. The collagen volume ratio was significantly increased in AM-treated knees by 39% and 31% at 0.1 μg and 3 μg AM, respectively, compared with control knees (Figures 8 and 9). The effects of AM on these pathological tissue changes, however, were not observed in knees treated with low-dose AM.

### Cytokines

**Figure 10:**
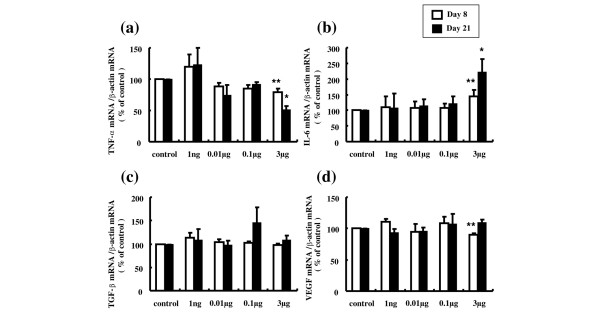
**Effect of adrenomedullin on cytokine mRNA expression linked to antigen-induced arthritis**. Expression levels of TNFα, IL-6, transforming growth factor beta (TGFβ), and vascular endothelial growth factor (VEGF) mRNA in the infrapatellar fat pads were determined by real-time quantitative PCR. **(a) **Adrenomedullin (AM) treatment reduced TNFα mRNA expression in a dose-dependent manner. Daily intra-articular injections of 3 μg AM significantly suppressed the TNFα mRNA level by 21% and 49% at day 8 and day 21, respectively. **(b) **AM increased IL-6 mRNA expression in a dose-dependent manner. Daily intra-articular injections of 3 μg AM significantly increased the IL-6 mRNA level by 45% and 121% at day 8 and day 21, respectively. **(c) **AM treatment did not alter the TGFβ mRNA level. **(d) **Although the VEGF mRNA level was suppressed by 10% at 3 μg AM on day 8, a dose-dependent effect of AM on VEGF mRNA expression was not observed. Open and closed columns represent the data at day 8 (*n* = 5 in each group) and day 21 (*n* = 3 in each group), respectively. Data expressed as the mean ± standard error of the mean. *P < 0.05 and **P < 0.01, compared with contralateral knees

To elucidate the mechanism of the anti-inflammatory effects of AM in inflamed joints, we investigated the effect of AM on cytokine mRNA expression linked to AIA. Treatment with AM reduced TNFα mRNA expression in a dose-dependent manner. Daily intra-articular injections of 3 μg AM significantly suppressed the TNFα mRNA level by 21% and 49% at day 8 and day 21, respectively, compared with controls (Figure 10a). In contrast, AM dose-dependently increased IL-6 mRNA expression. Daily intra-articular injections of 3 μg AM significantly increased the IL-6 mRNA level by 45% and 121% at day 8 and day 21, respectively, compared with controls (Figure 10b). Although the VEGF mRNA level was suppressed by 10% at 3 μg AM on day 8, we did not observe a dose-dependent effect of AM on VEGF mRNA expression (Figure 10d). AM treatment did not significantly alter the TGFβ mRNA level (Figure 10c).

## Discussion

In the present study we have shown that daily injections of AM into the knee joint spaces of rabbits with AIA ameliorated the inflammatory response associated with the disease. Treatment with AM reduced joint swelling, and reduced the expression of TNFα mRNA, edematous changes and the number of infiltrating inflammatory cells in the synovial tissue. To the best of our knowledge, this is the first report to show the effects of daily intra-articular injections of AM in rabbits with AIA.

We observed that AM suppressed joint swelling (Figures 2 and 3). Histologically, AM treatment reduced edematous changes and increased the ratio of connective tissue in the infrapatellar fat pad (Figures 7, 8 and 9). A previous study showed that TNFα induced cytoskeletal reorganization of endothelial cells and increased endothelial permeability by stimulating TNF receptors 1 and 2 [17]. In addition, TNFα facilitates the ability of VEGF to promote excessive vascular permeability [18]. TNFα also suppresses the expression of matrix genes and the induction of connective tissue growth factor by TGFβ during the wound healing response [19]. TNFα therefore aggravates edematous changes and suppresses the fibrotic response of the tissue. Moreover, AM was shown to reduce endothelial hyperpermeability induced by hydrogen peroxide, thrombin, and *Escherichia coli *hemolysin [20].

Two research groups reported recently that AM signaling deficiency in mice resulted in midgestation death and massive edema. The cause of this edema was shown to be a result of fragility and hyperpermeability of blood vessels in one group and to be a failure of lymphatic vessel growth in the other [21,22]. The evidence from these studies suggests that AM plays an important role in preventing edema. From these observations, we speculate that AM not only suppresses the production of TNFα, but also directly and indirectly inhibits edematous changes in the inflamed joint.

Although RA is a chronic and systemic inflammatory disorder of unknown etiology, TNFα has been shown to play a central role in the pathogenesis of RA [1,2,23]. TNFα stimulates the proliferation of synovial cells and the production of matrix metalloproteinases by chondrocytes and synovial cells, and induces the release of other proinflammatory cytokines, leading to joint destruction [23,24]. We have shown that daily injections of AM into the knee joint spaces of rabbits with AIA suppressed the expression of TNFα mRNA in the synovial tissue in a dose-dependent manner (Figure 10a). It has been reported that AM suppressed the secretion of TNFα from lipopolysaccharide-stimulated RAW 264.7 macrophages and NR8383 macrophages [4-6]. Because the major source of TNFα in inflamed synovial tissue of RA is due to macrophages [25], it is plausible that AM suppresses the production of TNFα from activated macrophages in inflamed synovial tissue.

On the contrary, we found that AM increased IL-6 mRNA expression in the synovial tissue (Figure 10b). Our results agree with previous findings on the effects of AM on IL-6 production. AM is reported to augment the production of IL-6 from NR8383 cells and Swiss 3T3 fibroblast cells stimulated with lipopolysaccharide or cytokines [4,26]. Several observations support the concept that IL-6 is an anti-inflammatory cytokine [27]. IL-6 has been shown to have a suppressive effect on TNFα and IL-1β production in peripheral blood mononuclear cells and exerts its anti-inflammatory effects in hepatitis by reducing the production of TNF [28,29]. Our results therefore lead us to speculate that the mechanism involved in the anti-inflammatory effects of AM is related to suppression of TNFα in inflamed synovial tissue directly or through IL-6 production.

Overproduction of IL-6 has been observed and is known to cause unfavorable clinical symptoms in immune-inflammatory diseases such as RA. Overproduction of IL-6 induces the production of rheumatoid factors and increases antibody levels, the platelet count, C-reactive protein levels, and serum amyloid A protein levels in RA [30]. Treatment with a humanized anti-IL-6 receptor antibody has also been shown to reduce RA disease activity [30,31]. The effect of AM on IL-6 production might therefore be an undesirable adverse effect in RA therapy. Plasma AM levels have been reported to increase with RA disease activity and in the acute or flare phase of myocardial infarction and sepsis [10,11,32,33]. Recent studies have shown that AM administration in the acute phase reaction of several disease models produced significant protective effects in organs against inflammation and oxidative stress [34-36]. Miyashita and colleagues reported that AM administration to prevent ischemic brain damage in mice less than 72 hours after the ischemic event showed significant therapeutic effects, whereas AM administration more than 72 hours after stroke onset produced no significant therapeutic effects [37].

From these observations and our study findings, we speculate that the effects of AM may be dependent on the tissue environment and the disease state; that is, the role and effects of AM in inflammation may change during the inflammatory process. AM acts as a strong anti-inflammatory agent in the acute or flare phase of inflammation, but in the chronic phase of inflammation AM may act not only as an anti-inflammatory agent but also as a proinflammatory agent. It is therefore important to consider the time of administration, the route of administration and the dosage schedule of AM in the treatment of RA.

## Conclusion

In the present study, the effects of daily intra-articular injections of AM into the knees of rabbits with AIA were examined. The results suggest that AM suppresses the inflammatory response in inflamed joints by inhibiting the expression of TNFα mRNA and increasing IL-6 mRNA level.

Although AM may have anti-inflammatory properties, the effect of AM on IL-6 production in inflamed synovial tissue might be an undesirable adverse effect in RA therapy. Further research is necessary to investigate the drug effects, the time of administration and the dosage schedules of intra-articular injection of AM in the treatment of RA.

## Abbreviations

AIA: antigen-induced arthritis; AM: adrenomedullin; H & E: hematoxylin and eosin; IL: interleukin; PCR: polymerase chain reaction; RA: rheumatoid arthritis; RT: reverse transcriptase; TGFβ: transforming growth factor beta; TNF: tumor necrosis factor; VEGF: vascular endothelial growth factor.

## Competing interests

The authors declare that they have no competing interests.

## Authors' contributions

TO and KM had full access to all of the study data and take full responsibility for the integrity of the data and the accuracy of the data analysis. EC and HH conceived the study, and participated in the study design. TS helped to develop the animal model and draft the manuscript. TF helped to carry out real-time PCR and perform statistical analyses. YA performed the histological evaluation. KK measured the level of AM in plasma and participated in the study design.
